# Brain abscess and hereditary hemorrhagic telangiectasia

**DOI:** 10.1590/0004-282X-ANP-2021-0389

**Published:** 2022-03-13

**Authors:** Leonardo Furtado FREITAS, Márcio Luís DUARTE, Eduardo Carvalho MIRANDA

**Affiliations:** 1 Universidade Federal de São Paulo, Departamento de Radiologia, São Paulo SP, Brazil. Universidade Federal de São Paulo Departamento de Radiologia São Paulo SP Brazil; 2 Universidade Federal de São Paulo, Departamento de Saúde Baseada em Evidências, São Paulo SP, Brazil. Universidade Federal de São Paulo Departamento de Saúde Baseada em Evidências São Paulo SP Brazil; 3 Rede MaterDei de Saúde, Departamento de Neurorradiologia, Belo Horizonte MG, Brazil. Rede MaterDei de Saúde Departamento de Neurorradiologia Belo Horizonte MG Brazil

**Figure 1.**
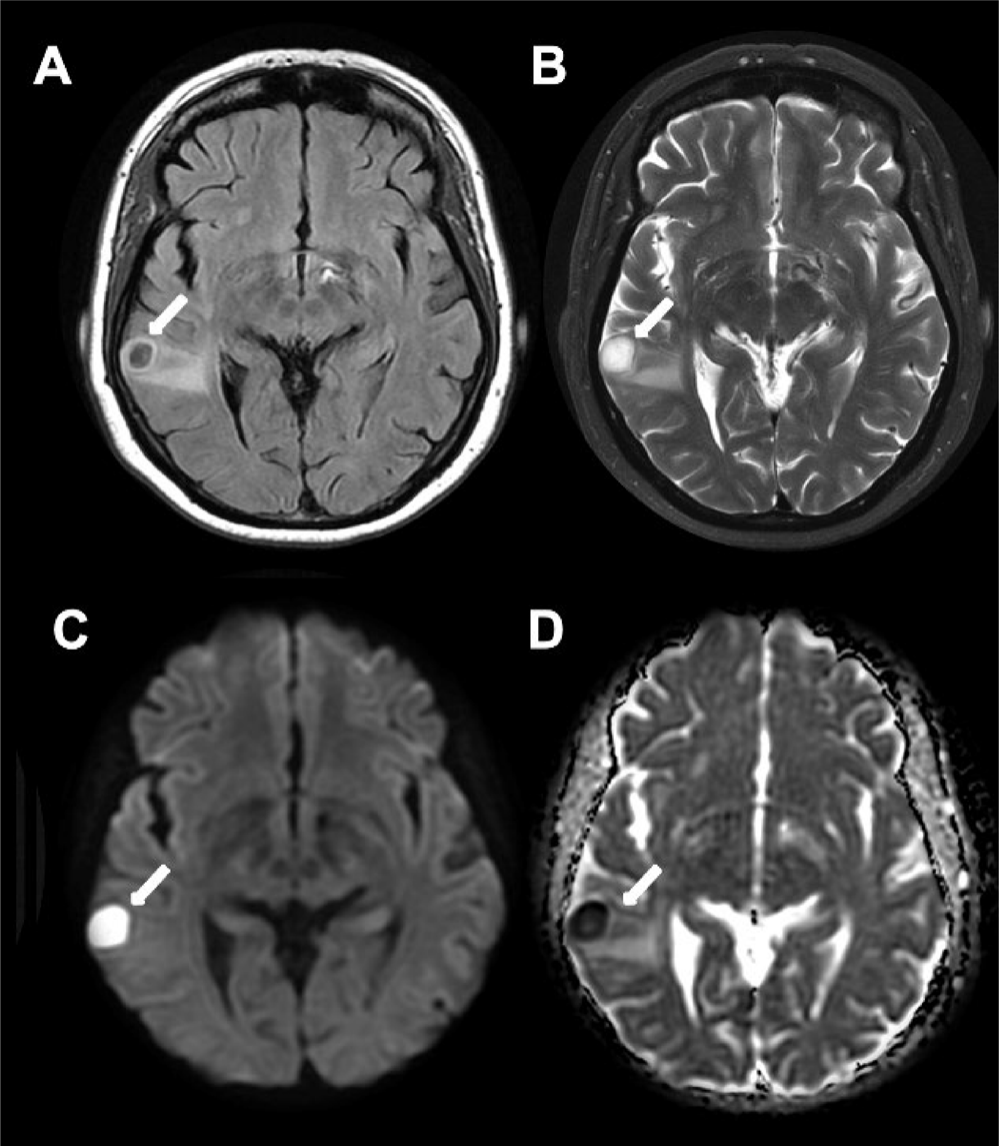
Brain MRI in axial FLAIR (A), T2-weighted (B), B1000 diffusion (C), and ADC map (D). A pyogenic abscess surrounded by vasogenic edema (arrows) in the right superior temporal gyrus is detected.

**Figure 2.**
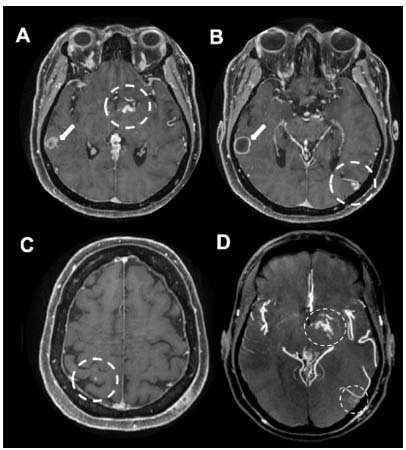
Brain MRI in axial, post-gadolinium volumetric T1 (A to C) and MIP (D) sequences. A pyogenic abscess surrounded by vasogenic edema (arrows) in the right superior temporal gyrus is detected (arrows in A and B). Dashed circles of images A to D highlight multiple tiny vascular malformations in both cerebral hemispheres (capillary malformations and microMAVs), more conspicuous in the MIP sequence.

**Figure 3.**
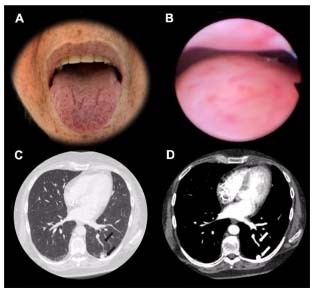
Face ectoscopy (A) and rhinoscopy exam (B); CT of the chest in the lung (C) and soft tissue (D) windows. Other systemic changes in the spectrum of the syndrome: multiple tiny cutaneous telangiectasia on the face, tongue, and labial mucosa (A), as well as on the nasal mucosa (B) and pulmonary vascular malformation compatible with arteriovenous malformation (AVM) (arrows on C and D).

A 58-year-old woman presented speech impairment, mental confusion, and left hemiparesis after being found unconscious. Brain magnetic resonance imaging (MRI) showed pyogenic abscess and multiple vascular malformations in the cerebral hemispheres (Figures 1 and 2), resulting in suspicion of Rendu-Osler-Weber disease, i.e., hereditary hemorrhagic telangiectasia (HHT). Chest computed tomography (CT) revealed pulmonary arteriovenous malformation. Ectoscopy detected multiple telangiectasia in the lips, tongue, face, and nasosinusal mucosa (Figure 3). HHT is a rare systemic fibrovascular dysplasia (prevalence rate: 1:50000-100000)^1^, and brain abscess is an acute and easily forgotten complication that occurs in 1% of patients with considerable mortality, i.e., death rate of 40%^2^.
